# Concurrent validity of self-rating scale of self-directed learning and self-directed learning instrument among Italian nursing students

**DOI:** 10.1186/s12912-016-0142-x

**Published:** 2016-03-21

**Authors:** Lucia Cadorin, Su-Fen Cheng, Alvisa Palese

**Affiliations:** CRO Aviano National Cancer Institute, Via F. Gallini, 233081 Aviano, Pordenone Italy; College of Nursing, National Taipei University of Nursing and Health Sciences, 365, Ming-Te Rd, Peitou District, Taipei, 11219 Taiwan, ROC; University of Udine, Viale Ungheria, 20-33100 Udine, Italy

**Keywords:** Self Directed Learning, Concurrent validity, Nursing students, Learning evaluation

## Abstract

**Background:**

Self-Directed Learning develops when students take the initiative for their learning, recognising needs, formulating goals, identifying resources, implementing appropriate strategies and evaluating learning outcomes. This should be seen as a collaborative process between the nurse educator and the learner. At the international level, various instruments have been used to measure Self-Directed Learning abilities (SDL), both in original and in culturally-adapted versions. However, few instruments have been subjected to full validation, and no gold standard reference has been established to date. In addition, few researchers have adopted the established tools to assess the concurrent validity of the emerging new tools. Therefore, the aim of this study was to measure the concurrent validity between the Self-Rating Scale of Self-Directed Learning (SRSSDL__Ita_) – Italian version and the Self-Directed Learning Instruments (SDLI) in undergraduate nursing students.

**Methods:**

A concurrent validity study design was conducted in a Bachelor level nursing degree programme located in Italy. All nursing students attending the first, second or third year (*n* = 428) were the target sample. The SRSSDL__Ita_, and the SDLI were used. The Pearson correlation was used to determine the concurrent validity between the instruments; the confidence of intervals (CI 95 %) bias-corrected and accelerated bootstrap (BCa), were also calculated.

**Results:**

The majority of participants were students attending their first year (47.9 %), and were predominately female (78.5 %). Their average age was 22.5 ± 4.1. The SDL abilities scores, as measured with the SRSSDL__Ita_ (min 40, max 200), were, on average, 160.79 (95 % CI 159.10–162.57; median 160); while with the SDLI (min 20, max 100), they were on average 82.57 (95 % CI 81.79–83.38; median 83). The Pearson correlation between the SRSSDL__Ita_ and SDLI instruments was 0.815 (CI BCa 95 % 0.774–0.848), (*p* = 0.000).

**Conclusions:**

The findings confirm the concurrent validity of the SRSSDL__Ita_ with the SDLI. The SRSSDL__Ita_ instrument can be useful in the process of identifying Self-Directed Learning abilities, which are essential for students to achieve the expected learning goals and become lifelong learners.

## Background

Self-Directed Learning (SDL) is a core concept in nursing education where the aim is to design and implement interventions reflecting the principles of adult education [1]. SDL has been identified as an approach to learning, as well as a core professional standard for all healthcare professionals [2].

SDL is described as a process in which the student or the healthcare professional determines his/her learning aims, with or without the help of the nurse educators, and chooses to implement appropriate methods or strategies to achieve the learning aims identified, assessing the achieved learning outcomes [3]. In accordance with Knowles’ SDL conceptual definition, which is still the most reported in nursing literature [1, 4–10], SDL is based on seven core components: 1) the nursing educator as a facilitator, 2) the identification of learning needs, 3) the development of learning aims, 4) the identification of appropriate resources, 5) the implementation of the learning process, 6) the commitment to a learning contract, and 7) the evaluation of learning outcomes [11]. In accordance with Garrison’s conceptual model, SDL includes attributes and skills integrating a complex process of external management, internal monitoring, motivation and factors associated with learning in an educational context [1, 12].

In recent years, SDL has received increased attention in the context of higher education [2]. Higher SDL abilities have been associated with increased curiosity, critical thinking, quality of understanding, retention, recall, and competence, as well as better decision-making [8, 10] and significant learning [13]. SDL has also been associated with increased motivation, self-confidence and independence, interpersonal communication abilities, which are well recognised essential components of nurses’ professional development [7]. Furthermore, SDL abilities have been associated with increased flexibility, clinical competence and the ability to deal with the emerging challenges of the healthcare context [4]. Therefore, nurse educators and students are recommended to improve their understanding of SDL abilities and to identify strategies required to improve SDL abilities [14]. Monitoring SDL abilities can help the student to identify their own needs, and can also help educators to measure the effectiveness of the SDL strategies adopted.

At the international level, various instruments measuring SDL abilities have been developed in original and culturally adapted versions. In accordance with its publication date, the Self-directed Learning Readiness Scale has been acknowledged as the first instrument developed (SDLRS: [2, 15–18]), followed by the Oddi Continuing Learning Inventory (OCLI: [19]), the Self-Rating Scale of Self-Directed Learning (SRSSDL: [20, 21]), and the Self-Directed Learning Instrument (SDLI: [5, 8, 22]). The psychometric properties of each instrument are reported in Table 1.

**Table 1:** Psychometric characteristics of SDL instruments

Instrument	Source	Country^a^	Description	Participants	Psychometric Indices
Self-Directed Learning Readiness Scale (SDLRS)	Guglielmino, 1977 [15]	US	Eight factors: openness to learning opportunities, self-concept as an effective learner, initiative and independence in learning, informed acceptance of responsibility for one’s own learning, love of learning, creativity, positive orientation to the future, ability to use basic study skills, and problem-solving skills Total items: 57 items 5-point Likert scale Score range: 57–285	307 students (subsequently validated in nursing students: [12, 16, 18, 41])	Content validity: 3 rounds of Delphi study Internal consistency: α = .87 Construct validity: EFA explained variance: 48 % Concurrent validity: SDLRS vs. observed behaviours of the students – Self-Esteem *r* = .39, *p*. <0.01; Self-Efficacy *r* = .58, *p*. <0.01; Attitude *r =* .60, *p*. <0.01 [33]
Self-Directed Learning Readiness Scale (SDLRS)	Fisher et al., 2001 [16]	AU	Three factors: self-management, desirefor learning, and self-control Total items: 40 5-point Likert scale Score range: 40–200	201 nursing students	Content validity: 2 rounds of Delphi study Internal consistency: α = .924 Construct validity: EFA explained variance: 36.4 % Concurrent validity: None reported
Turkey version of Self-Directed Learning Readiness Scale (SDLRS__TU_)	Kocaman et al., 2006 [17]	TR	Three factors: self-management, desire for learning, and self-control Total items: 40 5-point Likert scale Score range: 40–200	50 nursing students	Content validity: Translation from English to Turkish Internal consistency: α = .94 Construct validity: Known-groups technique *t* = 7.808 (*p* = 0.000) Concurrent validity: None reported
Self-Directed Learning Readiness Scale (SDLRS)	Fisher and King, 2010 [18]	AU	Three factors: self-management, desire for learning, and self-control Total items: 40 5-point Likert scale Score range: 40–200	227 nursing students	Content validity: None reported Internal consistency: α = .87 Construct validity: CFA sub scale: Self management RMSEA = .039, GFI = .960, GFI-AGFI = .023, CFI = .971, SRMR = .039, Desire for learning RMSEA = .024, GFI = .971, GFI-AGFI = .020, CFI = .993, SRMR = .032, Self-control RMSEA = .054, GFI = .951, GFI-AGFI = .028, CFI = .930, SRMR = .031 Concurrent validity: None reported
Self-Directed Learning Readiness Scale (SDLRS)	Williams and Brown, 2013 [2]	US	Three factors: self-management, desire for learning, and self-control Total items: 40 5-point Likert scale Score range: 40–200	233 undergraduate paramedics	Content validity: None reported Internal consistency: α = .90 Construct validity: CFA: RMSEA (90 % confidence interval (CI) = 0.102 (0.087, 0.116), AGFI = 0.815, CFI = 0.817, TLI = 0.781, and SRMR = 0.078. The best fitting model was the four-factor 36-item and three-factor 29-item. Concurrent validity: None reported
Oddi Continuing Learning Inventory (OCLI)	Oddi et al., 1990 [19]	US	Three factors: motivational, effective and cognitive attributes: proactive drive versus reactive drive; commitment to learning versus apathy/aversion to learning and cognitive openness versus defensiveness Total items: 24 items 7 point Likert scale Score range: 24–168	256 registered nurses	Content validity: Panel of experts Internal consistency: α = .90 Construct validity: EFA explained variance: 45.7 % Concurrent validity: OCLI vs. three sub scale of the Adjective Checklist (ACL) [42]: Tendency to seek affiliation with others *r* = .26; Work productively and consciously toward a goal *r* = .53; Manifest initiative and high levels of aspiration *r* = .55.
Self-Rating Scale of Self-Directed Learning (SRSSDL)	Williamson, 2007 [20]	UK	Five factors: awareness, learning strategies, learning activities, evaluation, and interpersonal skills Total items: 60 5-point Likert scale Score range: 60–300	30 nursing students	Content validity: 2 rounds of Delphi study Internal consistency: α = .71–.79 Construct validity: Known-groups technique, data not reported Concurrent validity: None reported
Italian version of Self-Rating Scale of Self-Directed Learning (SRSSDL__Ita_)	Cadorin et al., 2013 [21]	IT	Eight factors: awareness, attitudes, motivation, learning strategies, learning methods, learning activities, interpersonal skills and constructing knowledge Total items: 40 5-point Likert scale Score range: 40–200	847 nurses, radiology technicians, nursing students and radiology technician students	Content validity: Translation and back-translation Internal consistency: α = .92 Construct validity: EFA explained variance 53.3 % and CFA^b^ > *χ* ^2^ (712) = 1104.273 with *p* < 0.001, RMSEA = 0.031 (lower bound 0.027; upper bound 0.054) with *p* = 1.00, SRMR = 0.055 Concurrent validity: None reported
Self-Directed Learning Instrument (SDLI)	Cheng et al., 2010 [5]	TW	Four factors: learning motivation, planning and implementing, self-monitoring, and interpersonal communication. Total items: 20 5-point Likert scale Score range: 20–100	1,072 nursing students	Content validity: 2 rounds of Delphi study Internal consistency: α = .91 Construct validity: CFA RMS = 0.04, RMSEA = 0.057, GFI = 0.94, AGFI = 0.92, NFI = 0.93) Concurrent validity: None reported
Self-Directed Learning Instrument (SDLI)	Shen et al., 2014 [8]	CN	Four factors: learning motivation, planning and implementing, self-monitoring, and interpersonal communication. Total Items: 20 5-point Likert scale Score range: 20–100	1,499 nursing students	Content validity: None reported Internal consistency: α = .91 Construct validity: EFA explained variance 53.3 % CFA: RMR = 0.028, RMSEA = 0.057, CFI = 0.930, GFI = 0.929, AGFI = 0.909, PGFI = 0.781, NFI = 0.905 Concurrent validity: SDI vs. SRSSDL Pearson .87 (*p* = .000)

^a^ *US* United States, *UK* United Kingdom, *IT* Italy, *TR* Turkey, *CN* China, *TW* Taiwan, *AU* Australia

^b^under publication

*Note*: *α* Cronbach’s Alpha Coefficient – Total scale, *r* Pearson’s Coefficient, *t t*-test, *EFA* Explorative Factor Analysis, *CFA* Confirmatory Factor Analysis, *RMSEA* Root Mean Square Error of Approximation, *SRMR* Standardised Root Mean Square Residual, *RMS* Standardised Residual, *RMR* Root Mean Square Residual, *CFI* Comparative Fit Indices, *PGFI* Parsimony Goodness-of-Fit Index, *AGFI* Adjusted Goodness of Fit Index, *GFI* Goodness of Fit, *NFI* Normed Fit Index, *ACL* Adjective Checklist comprised of the 300 adjectives commonly used to describe a person’s behavioural tendencies and attributes

All instruments have been validated in nursing students and with varied health-care populations using different methods. However, to date, a gold standard instrument has not yet been identified [23] and given the proliferation of instruments reflecting different approaches and theoretical assumptions, such as the Knowles’s theory, Garrison’s Model or Zimmerman’s SDL models [1, 24, 25], the concurrent validation of the instruments is recommended. Concurrent validity is a form of criterion validity and refers to the degree of correlation between two measurements of the same concept provided simultaneously [26, 27].

To date, only Guglielmino’s, Oddi’s and Cheng’s instruments have been subjected to a criterion-related validation based on concurrent validity [5, 15, 19]; in particular, only Cheng’s instrument has been compared with another SDL instrument [8]. However, the instrument adopted by Shen and colleagues (2014) to perform the criterion validation was not subjected to a complete validation as has been previously described in the literature [20]. Therefore, a lack of information remains in the field of concurrent validity of SDL instruments, which would be aimed at comparing the measures obtained with an instrument as a criterion, as the standard by which the measures are being judged or evaluated. Therefore, the purpose of our study was to establish the concurrent validity between SRSSDL and the SDLI used in undergraduate nursing students.

## Methods

### Study design

A concurrent validity study design was performed [27].

### Setting, sample and sampling

A Bachelor of Nursing Science Course (BNSc) located in Italy was chosen for participant recruitment. The Dean of the Degree gave the authorisation to approach the students after having discussed the research protocol. Thereafter nursing students attending the first, second or third years of the BNSc were invited to participate in the study. The eligibility criteria were: a) full-time students attending the BNS degree programme, and b) those willing to participate in the study. Students were invited to participate after having received appropriate information regarding the study aims and its procedures.

### Instruments

A questionnaire that included socio-demographic variables, the Self-Rating Scale of Self-Directed Learning – Italian version (SRSSDL__Ita_) and the Self-Directed Learning Instrument (SDLI) were used for data collection performed between September and October 2014.

### Self-Rating Scale of Self-Directed Learning (SRSSDL)

The SRSSDL, originally developed by Williamson and then validated in the Italian context [28], was adopted. The Italian version of the SRSSDL has demonstrated good internal consistency (Cronbach’s alpha [α] coefficient 0.92) [21]. The SRSSDL__Ita_ consists of 40 items distributed into eight factors: “Awareness” which includes seven items (α = 0.80); “Attitudes”, eight items (α = 0.77); “Motivation”, six items (α = 0.78); “Learning strategies”, five items (α = 0.78); “Learning methods”, four items (α = 0.67); “Learning activities”, four items (α = 0.68); “Interpersonal skills”, four items (α = 0.68); and “Constructing knowledge”, two items (α = 0.73).

These factors were identified according to the Knowles’s andragogical theory [25] and in accordance with the findings that emerged from Explorative Factor Analysis (EFA) [21] and were confirmed using Confirmatory Factor Analysis (CFA) (article submitted, under revision). The responses for each item were rated using a five-point Likert scale ranging from 1 for never to 5 for always. Therefore, the total score of the SRSSDL__Ita_ ranged from 60 to 300 [21]. This score indicated lower and higher levels of SDL abilities. The low scores indicates poor SDL abilities and the guidance of a nursing educator is needed for the student; high scores, on the contrary, indicates higher SDL abilities and an independent-learning student. The instrument (pencil–paper) takes around 15 min to complete.

### Self-Directed Learning Instrument (SDLI)

Cheng et al. (2010) developed the SDLI which has demonstrated good internal consistency (Cronbach’s total scale was α = 0.916) [5]. It is comprised of 20 items categorised into four domains: “Learning motivation”, six items (α = 0.80), “Planning and implementing”, six items (α = 0.86), “Self-monitoring”, four items (α = 0.78), and “Interpersonal communication”, four items (α = 0.76). These four domains are consistent with Knowles’s SDL theory [8]. The content validity was supported by a two-round Delphi study. The construct validity, internal consistency and reliability of the instrument were tested in a convenience sample of 1,072 nursing students in Taiwan.

A five-point Likert scale ranging from 1 for “strongly disagree” to 5 for “strongly agree” measured the level of self-directed learning with the SDLI. These scores depicted the individual student’s assessment of his/her own abilities of SDL. Therefore, “strongly disagree” depicts a very low level of (self-assessed) abilities, whereas “strongly agree” depicts a very high level of (self-assessed) abilities. The total possible score on the SDLI ranges from 20 to 100 [5, 22]. The SDLI (pencil–paper) takes around 10 min to be completed.

### Data collection process

Preliminarily, the English version of the SDLI [4] was translated into Italian after having obtained the author’s permission (21 June, 2014). The translation process was developed following Strainer and Norman’s [27] criteria aimed at achieving equivalence between the original version and the translated version of the instrument. Two independent translators were involved. They were informed with regard to the aims of the study, the process of validation and the underlying purpose of the translation, which was to guarantee cultural and language sensitivity [27]. Forward and backward translations were performed: the first English translator translated the SDLI [5] from the original English version into Italian. The second translator, working in a blinded fashion with respect to the original SDLI version, translated the Italian version obtained by the first translator into the English language. Both translators had adopted a language suitable to the context of healthcare and nursing due to their expertise in the healthcare sector and in the health professional continuing education field. Two researchers carried out the translation of the two versions independently at different times. Finally, the translators and researchers discussed the differences in culture and the meaning of words. The Italian version of the questionnaire was analysed by a selected group of six experts (nurse educators and PhD students engaged in nursing research) in order to confirm its face and content validity [29].

The English version instrument was then submitted for examination to its lead author [5] at the National Taipei College of Nursing, Taiwan, who confirmed its content validity. The author agreed that there was coherence between the original items and those that emerged in the process of translation, with the exception of the use of the term “education”. She suggested using the term “learning” instead of “education” given that the concept of “learning” is focused on the student, while the concept of “education” is focused on teachers/educators who have to provide the teaching strategies, methods and resources to the learners. This perspective embraces the new paradigm that has a stronger focus on student-centred learning, an active learning that promotes SDL [30–32].

The Italian version of the SDLI was then approved both by Italian and Taiwanese researchers and is available from the authors.

After having obtained the translated version of the SDLI, the two instruments (SDLI and SRSSDL__Ita_), comprising a total of 60 items, were both included in a questionnaire prepared for simultaneous student administration. Also included were the socio-demographic variables of the students: age, gender, marital status, secondary school attended before University enrolment, grade point obtained in secondary school (from 60, sufficient, to 100 which is the maximum score possible), previous university experience (yes/no), work experience before starting the BNSc (yes/no) and work experience attended (yes/no) during the BNSc.

### Ethical issues

The study was approved by the Internal Review Board of Udine University (September 2014). The informed consent of the participating students was also obtained after having provided information with regard to the aim of the study and the confidentiality of the data collected. Students were free to participate or not in the study and the questionnaire completion was considered an expression of the willingness to participate in this study. A researcher (LC) not involved in the education of the students distributed and collected the instrument. Completion time was approximately 25–30 min on average, and the participants’ anonymity was ensured.

### Data analysis

The statistical software SPSS version 22.0 for Windows was used for data analysis. Descriptive statistics were used for calculating frequencies, percentages, averages, standard deviations (±), median, skewness, kurtosis, and confidence intervals (CI) at 95 %. Measures of internal consistency (Cronbach’s alpha, α) were also searched for the total score and factors of the SRSSDL__Ita_ and SDLI instruments.

The Pearson correlation was used to estimate the concurrent validity between the SRSSDL__Ita_ and SDLI instruments. The confidence intervals – bias-corrected and accelerated (BCa) bootstrap (CI 95 % BCa) – was also calculated. The statistical significance was set as *p* <0.05.

## Results

### Participants

A total of 428 participants were enrolled (response rate of 90 %). The majority of participants were students attending their first year (47.9 %) and were predominately female (78.5 %). Their average age was 22.5 ± 4.1. Before enrolment in the BNSc, students had attended high school (81 %). A small proportion reported to have previous university experience, interrupting the attendance of the degree in a field other than nursing (20.6 %). One third of students (38.1 %) reported to have work experience before being enrolled in the BNSc. The full demographic profile of the participants is outlined in Table 2.

**Table 2:** Participants’ characteristics

Students	Total *n* = 428 (%)
1 year	205 (47.9)
2 year	111 (25.9)
3 year	112 (26.2)
Age, average (±)	22.0 ± 4.1
Gender	
Female	336 (78.5)
Male	90 (21.0)
Missing data	2 (0.5)
Marital Status	
Single	184 (43.0)
Married	10 (2.3)
Missing data	234 (54.7)
Secondary School	
High School	345 (81.0)
Technical school	81 (18.5)
Missing data	2 (0.5)
Grade Point out of 100, average (±)	76.7 ± 11.2
Previous University Experience(s)	
None	311 (72.6)
Completed (with graduation)	88 (6.8)
Abandoned	88 (20.6)
Missing data	–
Work Experience Before Starting BNSc	
Yes	163 (38.1)
No	253 (59.1)
Missing data	12 (2.8)
During the BNSc	
Yes	83 (19.4)
No	329 (76.9)
Missing data	16 (3.7)

### SDL abilities

As reported in Table 3, the SDL abilities scores, as measured with the SRSSDL__Ita_ (min 40, max 200), were on average 160.79 (95 % CI 159.10–162.57; median 160). Regarding the SDL measured with the SDLI (min 20, max 100), the score was, on average, 82.57 (95 % CI 81.79–83.38; median 83).

**Table 3:** SDL abilities as measured with SRSSDL__Ita_ and SDLI: Scores and internal consistency

	Average (CI 95 %)	Median	Skewness	Kurtosis	Range	α^a^
SRSSDL__Ita_ Factors (min–max score)						
Factor 1 Awareness (7–35)	28.52 (28.19–28.86)	29	−.429	−.047	17–35	0.828
Factor 2 Attitudes (8–40)	32.94 (32.58–33.29)	33	−.304	.137	19–40	0.694
Factor 3 Motivation (6–30)	24.17 (23.85–24.49)	24	−.246	−.313	13–30	0.806
Factor 4 Learning strategies (4–20)	15.08 (14.82–15.34)	15	−.237	.083	6–20	0.646
Factor 5 Learning methods (5–25)	20.37 (20.09–20.66)	20	−.158	−.177	10–25	0.846
Factor 6 Learning activities (4–20)	16.16 (15.91–16.40)	16	−.235	−.530	8–20	0.746
Factor 7 Interpersonal skills (4–20)	16.24 (15.99–16.50)	16	−.298	−.310	8–20	0.800
Factor 8 Construction knowledge (2–10)	7.28 (7.08–7.49)	8	−.537	−.137	2–10	0.903
Total score (40–200)	160.79 (159.10–162.57)	160	−.108	−.057	112–200	0.931
SDLI Factors (min–max score)						
Factor 1 Learning motivation (6–30)	25.85 (25.59–26.12)	26	−.456	−.218	17–30	0.752
Factor 2 Planning and implementing (6–30)	24.22 (23.91–24.54)	24	−.217	−.204	12–30	0.860
Factor 3 Self-monitoring (4–20)	16.62 (16.44–16.80)	16	.049	−.647	11–20	0.821
Factor 4 Interpersonal communication (4–20)	15.87 (15.67–16.09)	16	−.466	.925	5–20	0.640
Total score (20–100)	82.57 (81.79–83.38)	83	−.205	−.224	54–100	0.903

Missing items < 1 %

^a^Cronbach’s Alpha Coefficient

The values of skewness and kurtosis both in the SRSSDL__Ita_ and SDLI total scores and in the factors were calculated. The SRSSDL__Ita_ total score skewness was −.108, while for the SDLI, it was −.205. Negative values greater than .30 were reported in SRSSDL – “Awareness” (−.429), “Attitude” (−.304) and “Construction knowledge” (−.537) factors. In the SDLI instrument, negative values were instead reported as “Learning motivation” (−.446), and “Interpersonal communication” (−.466) factors. No value was greater than +1 or less than −1.

The SRSSDL__Ita_ kurtosis total score was −.057, while with the SDLI instrument, it was −.224. Negative values greater than −.30 were reported in SRSSDL__Ita_ under the “Motivation” (−.313), “Learning activities” (−.530), and “Interpersonal skills” (−.310) factors, while in the SDLI, negative values were found in the “Self monitoring” (−.647) factor. Positive values greater than .30 have emerged in the SDLI “Interpersonal communication” (.925) factor. No value was greater than +1 or less than −1.

### Concurrent validity

**Fig. 1:**
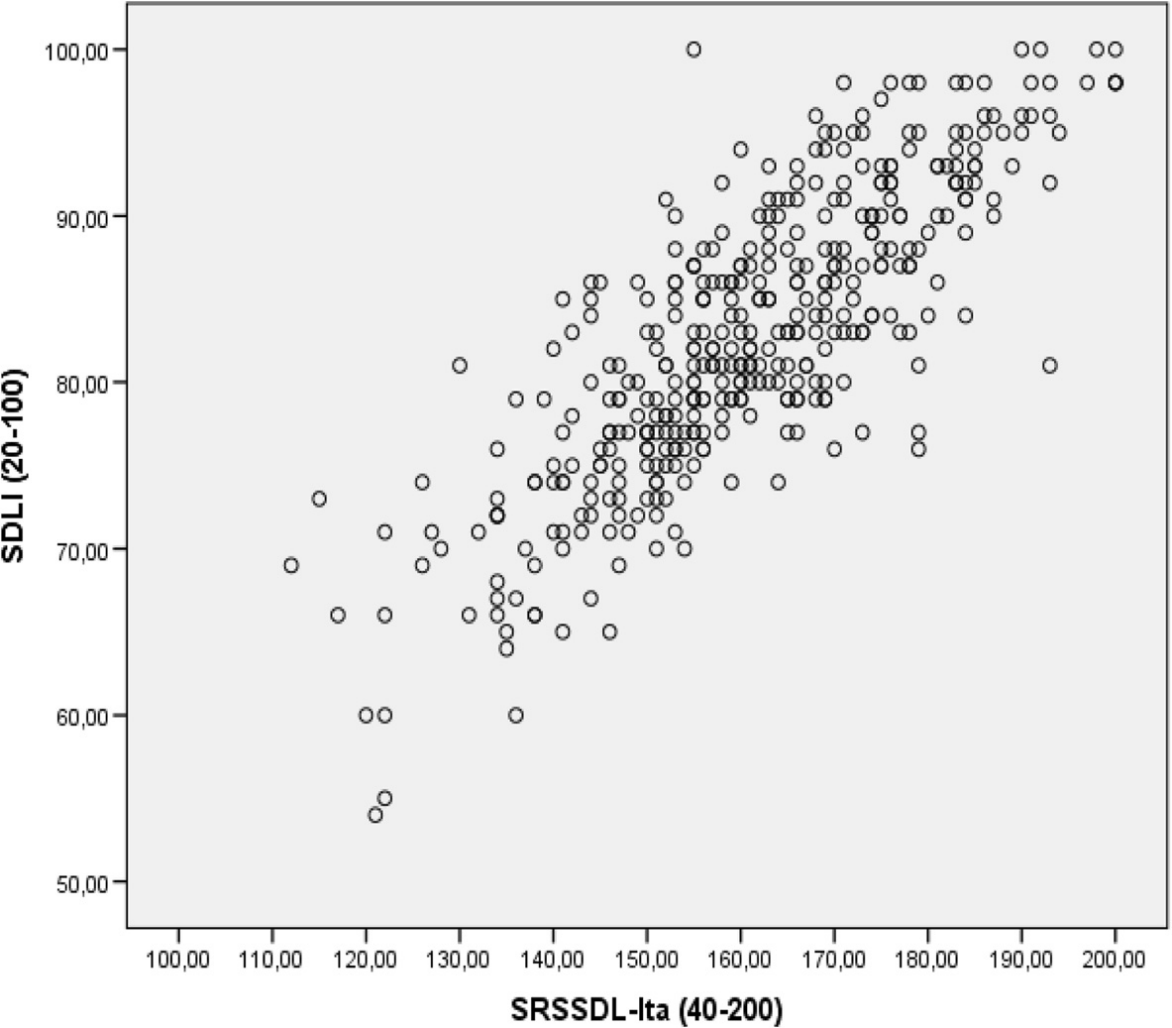
SRSSDL__Ita_ and SDLI score correlations

The Pearson correlation between the SRSSDL__Ita_ and SDLI instruments was 0.815 (CI BCa 95 % 0.774–0.848), (*p* = 0.000) as reported in Fig. 1. Therefore, some 66.4 % of the variance was common between the instruments, from 57.5 to 71.9 %.

## Discussion

This is the second study, to our knowledge, that has evaluated the concurrent validity of two instruments in the field of SDL measurement in nursing education. Shen and colleagues conducted the first study, where the concurrent validity of the SRSSDL tool, in its original version, was established with the SDLI tool [8]. The authors reported a Pearson’s coefficient of .87 (*p* = .000). However, the SRSSDL version used for establishing concurrent validity was not fully validated. In addition, previously, few studies reported concurrent validity [19, 33] between instruments (OCLI vs. ACL and SDLRS vs. observed behaviours of the students) measuring different constructs.

A group of nursing students pursuing their bachelors level nursing degree in Italy were involved. Their demographic profile was in line with that previously documented in other studies performed in the Italian context [34, 35], and their size was appropriate with respect to the number of items included in each instrument. The ratio of one-item/10 participants was considered congruent with the target sample size established beforehand according to the recommendations stated by Pett and colleagues [36].

SDL abilities, as self-evaluated by students, were high for both instruments, leading to higher values in their internal consistency. Both skewness and kurtosis values were between −1 and +1, suggesting that the items were within the limits of a normal distribution [37]. However, different shapes of curves have emerged from the measures obtained by SRSSDL__Ita_ and SDLI instruments. Positive skewness indicates a tail to the right and negative skewness values indicates a tail to the left [37]. Greater negative skewness has been reported with the SDLI instrument (−.205), while the SRSSDL’s total skewness score was −.108. These values highlight the differences between the average and median (in the negative skewness, the average < the median), as well as the presence of extreme values that influence the average of the different SDL abilities levels among students [37].

With regard to the kurtosis, given that values > 1 indicate that the distribution tends to be pointed and values < 1 indicate that the distribution tends to be flat [37], in general more flattened values emerged in the SDLI instrument (−.224) compared with the SRSSDL__Ita_ total score (−.057). As for the skewness values, kurtosis values also show that SDL abilities levels were not assessed homogeneously among the instruments. In addition, positive kurtosis values have been reported in SDLI “Interpersonal communication” (.925), while negative values has been reported in the similar SRSSDL__Ita_ factor “Interpersonal skills” (−.310), therefore reflecting a divergence in the construct measured.

According to Knowles [25] the process of SDL covers the following domains: learning needs or learning motivation, resources, goals, plans and activities, evaluation, and communication skills. The majority of instruments documented in the literature are based on an andragogical model, which, according to Knowles’ [25] definition, would also cover the two instruments used in this study – the SRSSDL and the SDLI [5, 21]. Therefore, high correlation coefficients between the instruments using the same theory of SDL were expected.

From the findings, the correlation between the two instruments was *r* 0.815 (*p* = 0.000), and therefore some 66.4 % of the variance was common. According to Polit and Beck’s guidelines, the correlation was high and the common variance explained was good. Previously, in the Shen et al. study, the correlation between SDLI and SRSSDL (original version by Williamson) was higher (*r* 0.876) [8, 38]. Given that the instruments were developed and validated in different cultures (European [UK and Italy] and Taiwanese, respectively), reflecting a different commonality of meaning, customs and rules shared by a certain group of people and setting a complex framework for learning and development [38], the findings are appreciable and suggest that the instruments measure the same construct with different factor numbers and items, both at qualitative and quantitative levels. Little attention to date has been given to the relationship between cultures and learning, especially in the field of SDL [39]; therefore, researchers working in different cultures are encouraged to share instruments and test their validity as the first step in developing international research networks.

This study offers a contribution also at the practical level. Promoting self-directed learning in higher education is an important graduate quality to develop in students entering professional practice. This is particularly important for nursing graduates who will work in complex and challenging health environments and must be responsible lifelong learners. Having validated tools to measure students’ SDL capabilities is important for educators so that they can evaluate how learners advance through stages of increasing self-direction.

The SRSSDL_-Ita_ is an instrument capable of offering feedback with regard to the SDL abilities of the learner. It may contribute to a) promoting and increasing awareness among students regarding their abilities with SDL and of their responsibility and autonomy in learning processes; b) identifying students with low SDL abilities and therefore at increased risk of experiencing difficulty in the university setting where independence in learning in expected; c) prompting students to reflect on their own learning methods and strategies, and in searching support when needed; d) identifying learning problems and needs, implementing strategies to enhance SDL abilities and evaluation and monitoring over time their effectiveness, and not lastly e) supporting educators in developing and evaluating SDL programmes and in designing new curriculum for BNSc courses or Masters Degree.

## Conclusions

This is the first study that evaluates the concurrent validity between two instruments developed in different cultures, while evaluating SDL abilities in the field of nurse education. Establishing the concurrent validity between two instruments developed in different cultures may help educators to develop international research projects based on common instruments, to compare findings and to test the effectiveness of different educational strategies implemented in different cultural contexts.

In accordance with the findings, the validity of the SRSSDL has been established in terms of measuring the SDL abilities of nursing students. Further psychometric evaluation of the SRSSDL, such as responsiveness of the scale to change, aimed at predicting how and why SDL abilities in nursing students change over time, will allow for the development of a more robust instrument, and therefore, increasing confidence in its validity when used in other studies [2, 40].

## Abbreviations

(Table) α: Cronbach’s Alpha Coefficient
ACL: Adjective Checklist
AGFI: Adjusted Goodness of Fit Index
BCa: bias-corrected and accelerated
BNSc: Bachelor of Nursing Science Course
CFA: Confirmatory Factor Analysis
CFI: Comparative Fit Indices
EFA: Explorative Factor Analysis
GFI: Goodness of Fit
NFI: Normed Fit Index
OCLI: Oddi Continuing Learning Inventory
PGFI: Parsimony Goodness-of-Fit Index
*r*: Pearson’s Coefficient
RMR: Root Mean Square Residual
RMS: Standardized Residual
RMSEA: Root Mean Square Error of Approximation
SDL: Self-directed learning
SDLI: Self-Directed Learning Instrument
SRMR: Standardized Root Mean Square Residual
SRSSDL: Self-Rating Scale of Self-Directed Learning
SRSSDL-_Ita_: Self-Rating Scale of Self-Directed Learning, Italian version
t: *t*-test
